# Chronically dysregulated NOTCH1 interactome in the dentate gyrus after traumatic brain injury

**DOI:** 10.1371/journal.pone.0172521

**Published:** 2017-03-08

**Authors:** Noora Puhakka, Anna Maria Bot, Niina Vuokila, Konrad Jozef Debski, Katarzyna Lukasiuk, Asla Pitkänen

**Affiliations:** 1 Department of Neurobiology, A. I. Virtanen Institute for Molecular Sciences, University of Eastern Finland, Kuopio, Finland; 2 The Nencki Institute of Experimental Biology, Polish Academy of Sciences, Warsaw, Poland; University of Modena and Reggio Emilia, ITALY

## Abstract

Traumatic brain injury (TBI) can result in several dentate gyrus-regulated disabilities. Almost nothing is known about the chronic molecular changes after TBI, and their potential as treatment targets. We hypothesized that chronic transcriptional alterations after TBI are under microRNA (miRNA) control. Expression of miRNAs and their targets in the dentate gyrus was analyzed using microarrays at 3 months after experimental TBI. Of 305 miRNAs present on the miRNA-array, 12 were downregulated (p<0.05). In parallel, 75 of their target genes were upregulated (p<0.05). A bioinformatics analysis of miRNA targets highlighted the dysregulation of the transcription factor NOTCH1 and 39 of its target genes (NOTCH1 interactome). Validation assays confirmed downregulation of miR-139-5p, upregulation of *Notch1* and its activated protein, and positive enrichment of NOTCH1 target gene expression. These findings demonstrate that miRNA-based transcriptional regulation can be present at chronic time points after TBI, and highlight the NOTCH1 interactome as one of the mechanisms behind the dentate gyrus pathology-related morbidities.

## Introduction

Each year ~1.5 million people in the United States and 2.5 million in Europe suffer traumatic brain injury (TBI) [1–3]. Some data suggest that life-compromising functional impairments develop in up to 43% of injured patients [2]. According to the World Health Organization, TBI will surpass many other diseases as the major cause of death and disability by the year 2020 http://www.who.int/violence_injury_prevention/road_traffic/activities/neurotrauma/en/). Thus, there is an urgent need to identify the molecular mechanisms of the post-TBI aftermath that could serve as treatment targets to improve recovery.

Current therapeutic interventions aimed at improving post-TBI recovery are focused on alleviating secondary brain damage during the first post-injury weeks [4–6]. Recent studies demonstrated that the development of secondary pathologies can continue for several weeks or even months, suggesting that the need for recovery-enhancing treatments could be more long-lasting than previously thought [7–11]. Very few studies, however, have investigated the molecular mechanisms of the evolution or maintenance of secondary pathologies beyond the first 2 weeks post-TBI [12–13]. Expression of micro RNAs (miRNAs) during the post-TBI aftermath was recently identified, and this promising category of transcriptional regulators has regulatory activities that persist for weeks to months beyond their expression [14–16].

Micro RNAs are small (19–22 nucleotides long) endogenous RNA molecules that regulate the expression of specific target genes at the post-transcriptional level via sequence-specific (seed) binding to the 3’ untranslated region (reviewed by [17]), which blocks protein translation or causes the degradation of target mRNAs [17]. Micro RNAs are highly conserved among species and there are no known sex differences [15, 18], which makes them appealing therapeutic targets in animal models of human disease. Moreover, miRNAs, especially circulating miRNAs, have demonstrated their potential as therapeutics as well as biomarkers for detecting the disease process and therapeutic response in humans, including brain cancers, Alzheimer’s disease, epilepsy, and multiple sclerosis [19–24]. In the acute stage after experimental TBI, dysregulated miRNAs reportedly target biologic processes, such as cellular functions (e.g., differentiation, proliferation), transcription, signal transduction, growth, protein modification, and response to stress, which can further impair recovery [14–15]. Surprisingly, despite this background, studies investigating brain miRNAs during the chronic post-TBI recovery phase to pinpoint miRNA-regulated molecular networks that could serve as therapeutic targets or biomarkers for TBI outcome have not yet been performed.

We hypothesized that chronically maintained post-TBI transcriptional changes are under miRNA regulation. Further, identification of those miRNAs and their target transcriptomes will reveal novel candidate pathways that could be modulated to enhance post-TBI recovery. With this aim, we induced TBI with lateral fluid-percussion injury, sampled the dentate gyrus at 3 months post-TBI, and assessed the expression of miRNAs using Exiqon miRNA arrays and their target mRNAs using an Affymetrix microarray. Our data pinpointed 12 miRNAs that were regulated at 3 months post-TBI. Further analysis indicated post-transcriptional regulation of the transcription factor NOTCH1 interactome present at chronic time points, which was confirmed with reverse transcription –polymerase chain reaction (RT-PCR) and immunohistochemistry.

## Materials and methods

**Fig 1:**
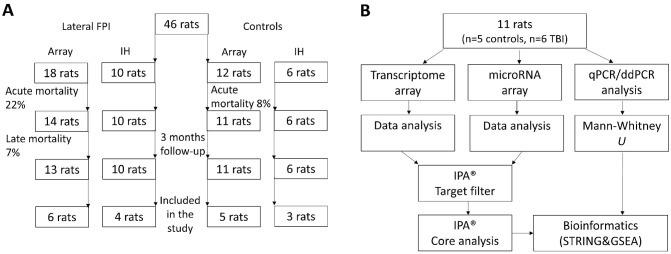
Flow-charts for the study design. Flow-charts showing the randomization of **(A)** 46 rats into the microRNA, transcriptomics, and immunohistochemical analysis and **(B)** 11 rats into the bioinformatics and validation experiments. Abbreviations: ddPCR, droplet digital PCR; FPI, fluid-percussion injury; GSEA, Gene Set Enrichment Analysis; IH, immunohistochemistry; IPA, Ingenuity Pathway Analysis; qPCR, quantitative PCR.

The study design is summarized in Fig 1.

### Animals

Eighteen adult male Sprague-Dawley rats [mean body weight at the time of injury, 326 g; range, 300-354g; Harlan Netherlands B.V., Horst, The Netherlands) were used in this study. The rats were individually housed in a controlled environment (temperature 22±1°C; humidity 50-60%; lights on from 07:00–19:00 h). Pellet food and water were provided *ad libitum*. All animal procedures were approved by the Animal Ethics Committee of the Provincial Government of The Southern Finland, and performed in accordance with the guidelines of the European Community Council Directives 2010/63/EU.

### Induction of traumatic brain injury with lateral fluid-percussion

The procedure for induction of lateral fluid-percussion injury (FPI) was described previously [25–26]. In brief, animals (n = 28) were anesthetized with an intraperitoneal (i.p.) injection of a solution (6 ml/kg) containing sodium pentobarbital (58 mg/kg), chloral hydrate (60 mg/kg), magnesium sulfate (127.2 mg/kg), propylene glycol (42.8%) and absolute ethanol (11.6%), and placed in a Kopf stereotactic frame (David Kopf Instruments, Tujunga, CA, USA). The skull was exposed with a midline skin incision and the periosteum extracted. The left temporal muscle was gently detached from the lateral ridge. A circular craniectomy (Ø 5 mm) was performed over the left parietal lobe midway between lambda and bregma, leaving the dura mater intact. The edges of the craniectomy were sealed with a modified Luer lock cap that was filled with saline while the calvaria was covered with dental acrylate (Selectaplus CN, Dentsply DeTrey GmbH, Dreieich, Germany). Lateral FPI was produced 90 min after the induction of anesthesia by connecting the rat to a fluid-percussion device (AmScien Instruments, Richmond, VA, USA) via a female Luer lock fitting. The mean severity of the impact was 3.23±0.01 atm (min 3.13—max 3.35 atm) in the group of animals used for the gene expression analysis (n = 18) and 3.15±0.02 atm (min 3.05—max 3.25 atm) in the group of animals used for immunohistochemistry (n = 10). Control animals (n = 18) received anesthesia and all surgical procedures without impact. From all surviving injured and sham-operated animals, we randomly selected 6 TBI and 5 control rats for gene expression analysis, and 4 TBI and 3 control rats for immunohistochemical analysis.

### Tissue sampling

#### Analysis of gene expression and miRNAs

At 3 months post-TBI, the rats were anesthetized with CO_2_ and decapitated with a guillotine. Brains were immediately removed from the skull and the left (ipsilateral) hippocampus was dissected out on ice (+4°C). Within 10 min after decapitation, the hippocampus was immersed into 1 ml of ice cold RNA*later* RNA Stabilization Reagent (#76106, Qiagen, Hilden, Germany) and stored at -20°C until further processed. For RNA extraction, the hippocampus was cut into transverse slices. The dentate gyrus and hilus were then dissected along the hippocampal fissure under a dissection microscope (SZ-630T, Delta Optical, Poland) using a scalpel blade.

#### Immunohistochemistry

At 3 months post-TBI, the rats were anesthetized with 4% isoflurane and decapitated with a guillotine. The brains were immediately removed from the skull and flash frozen in -70°C 2-methylbutane and stored at -70°C until processed. The brains were cut into 10-μm-thick sections using a cryostat (Leica CM3050 S, Leica Microsystems Nussloch GmbH, Germany) at -20°C. The tissue was mounted on slides and stored at -70°C until use.

### Isolation of total RNA

Total RNA (including miRNA) was isolated using the miRNeasy Mini kit (# 217004, Qiagen, Hilden, Germany) according to the manufacturer’s instructions (miRNeasy Mini Handbook 03/2013; http://www.qiagen.com/us/). The sample concentration, and contamination with protein or organic compounds, were determined using a NanoDrop 2000 spectrophotometer (Thermo, Fisher Scientific). The quality of the total RNA was further verified using the Agilent 2100 Bioanalyzer (Agilent Technologies, Santa Clara, CA, USA) using the manufacturer’s RNA 6000 Nano Kit (#5067–1511) for mRNA samples and Small RNA Analysis Kit (#5067–1548) for miRNA samples.

### miRNA profiling

The miRNA array profiling experiment was performed at Exiqon Services, Denmark, using the miRCURY LNA^™^ microRNA Array 7^th^ targeting all miRNAs for human, mouse, or rat registered in the miRBASE version 19.0 at the Sanger Institute. The data are available at Gene Expression Omnibus under the accession number GSE86615 (http://www.ncbi.nlm.nih.gov/geo/query/acc.cgi?acc=GSE86615). Final results after statistical analysis are presented in S1 Table. Sample numbers HC1, HC2, HC4, HC6, HC13 and HC15 refers to post-TBI rats and HC7, HC8, HC9, HC10 and HC18 to controls.

### Transcriptome profiling

GeneChip^®^ Rat Gene 1.1 ST arrays (# 901627, Affymetrix, Santa Clara, CA, USA) were used for mRNA profiling. Total mRNA (100 ng) was used for cDNA synthesis using an Ambion WT Expression Kit (# 4411974, Life Technologies). Hybridization, washing, and scanning were conducted according to Affymetrix guidelines for the GeneAtlas^™^ instrument. The data are available at Gene Expression Omnibus under the accession number GSE86579 (http://www.ncbi.nlm.nih.gov/geo/query/acc.cgi?acc=%20GSE86579). Final results after statistical analysis are presented in S2 Table. Sample numbers HC1, HC2, HC4, HC6, HC13 and HC15 refers to post-TBI rats and HC7, HC8, HC9, HC10 and HC18 to controls.

### Droplet digital PCR

Because the array profiling suggested a high interanimal variation within each experimental group and a small mean fold-change between the experimental groups, we applied droplet digital PCR (ddPCR) for more precise quantitation of miRNA copies in a given sample. First, the concentration of small RNAs in all samples was assessed using an Agilent small RNA assay (#5067–1548, Agilent Technologies). Thereafter, 10 ng of small RNA was reverse-transcribed to cDNA according to the manufacturer’s Droplet Digital PCR Applications guide (Bio-Rad Laboratories Inc., Hercules, CA, USA, www.bio-rad.com) using a Taqman reverse transcription kit (#4366596, Life Technologies). For every 20-μl PCR reaction, 1.33 μl of cDNA was mixed with 10 μl of Bio-Rad’s ddPCR supermix for probes, 1 μl of TaqMan^®^ Small RNA Assay (20x, #002289, Life Technologies), and 7.67 μl of nuclease-free water (#AM9939, Ambion, USA). Samples were loaded into the sample wells of the droplet generator cartridge (#1864008, Bio-Rad), and 70 μl of droplet generation oil for probes (#1863005, Bio-Rad) was added to the wells. Droplets were generated using a QX200 Droplet Generator (Bio-Rad) and applied to a 96-well plate (#951020303, Eppendorf, Hamburg, Germany). The plate was sealed with sealing foil (#1814040, Bio-Rad) using a PX1 PCR Plate Sealer (Bio-Rad) and placed in a PTC-200 Thermal Cycler (MJ Research) under the following conditions: 1 cycle at 95°C for 10 min; 40 cycles at 95°C for 15 s and at 60°C for 60 s; and finally, 1 cycle at 98°C for 10 min. Fluorescence of each droplet was measured with a QX100 Droplet Reader (Bio-Rad). To determine the copy number, data were analyzed with QuantaSoft software v1.7 (Bio-Rad). Each sample was run in duplicate. The number of positive droplets in the duplicates was combined and used for statistical analysis. A no template (nuclease-free water) control was added to each run.

### Quantitative RT-PCR

For quantitative RT-PCR, first-strand cDNA synthesis was performed with High Capacity RNA-to-cDNA Kit (#4387406, Applied Biosystems, Foster City, CA, USA) according to the user’s manual (http://tools.lifetechnologies.com/content/sfs/manuals/cms_047249.pdf) using 1 μg of total RNA. Quantitative RT-PCR was performed in a total volume of 20 μl using 12 ng (RNA equivalents) of cDNA as a template, gene-specific primers and probes (pre-validated TaqMan Gene Expression Assay for *Notch1*, ID: Rn01758633_m1, Applied Biosystems), and 1x TaqMan Gene Expression Master Mix (#4369016, Applied Biosystems). The following program was used in the PCR (StepOne Software v2.1, Applied Biosystems): 1 cycle (95°C, 10 min) and 40 cycles (95°C, 15 s; 60°C, 60 s) in a StepOnePlus^™^ Real-Time PCR System (Applied Biosystems). Data were normalized to *glyceraldehyde 3-phosphate dehydrogenase* (*Gapdh*) mRNA expression (pre-validated Taqman Gene Expression Assay for *Gapdh*; ID: Rn99999916_s1, Applied Biosystems). Each sample was run in triplicate. To each run a no template (nuclease-free water) control was added for both assays. For absolute quantitation, a 5-point standard curve was made using 75 ng, 37.5 ng, 18.8 ng, 9.4 ng, and 4.7 ng (RNA equivalents) of cDNA as a template for both assays.

### Immunohistochemistry

#### NOTCH1

For NOTCH1 immunohistochemistry, the sections were fixed with cold acetone (4°C) for 15 min on glass slides. Thereafter, they were washed three times with 0.02 M KPBS pH 7.4. To remove endogenous peroxidase activity, the sections were incubated in 1% H_2_O_2_ in 0.02 M KPBS at room temperature for 15 min. After washing three times in 0.02 M KPBS, nonspecific binding was blocked by incubating the sections in 10% normal goat serum (NGS) and 0.1% Triton X-100 in 0.02 M KPBS at room temperature for 2 h on glass slides. Sections were incubated for 3 d (4°C) in a primary antibody solution containing rabbit-polyclonal antibody raised against NOTCH1-Cleaved-Val1744 (1:100, #ab52301, RRID:AB_881726, Abcam, Cambridge, UK), 1% NGS, and 0.1% Triton X-100 in 0.02 M KPBS on slide glass. The sections were washed three times (2% NGS in 0.02 M KPBS) and incubated for 2 h with biotinylated anti-rabbit secondary antibody (1:200, Vector # BA-1000, Vector Laboratories, Burlingame, CA, USA) on glass slides at RT. Thereafter, sections were washed three times with 0.02 M KPBS and incubated with avidin-biotin solution (1:200, PK-4000, Vector Laboratories) on glass slides and washed again with 0.02 M KPBS. The secondary antibody was visualized with 0.1% 3’,3’-diaminobenzidine (Pierce Chemicals, Rockford, IL, USA) and 0.08% H_2_O_2_ in 0.02 M KPBS. Sections were washed three times in KPBS and once in 0.1 M PB. The slides were dried overnight at 37°C. Subsequently, the reaction product was intensified with osmium (OsO_4_) thiocarbohydrate according to the method of Lewis et al [27]. A control experiment was performed by omitting primary antibody from both control and TBI cases. Liu and colleagues [28] reported that the antibody is specific for NOTCH1 based on negative staining in NOTCH1-deficient (*Notch1* ^*−/−*^) mouse embryonic fibroblasts (MEFs).

To counterstain with cresyl violet, previously immunostained osmium-intensified sections were incubated in xylene for one week to remove coverslips, then immersed in 100% ethanol for 10 sec, followed by 96% ethanol for 30 sec, 70% ethanol for 30 sec, 50% ethanol for 30 sec, 1% cresyl violet (Merck, Darmstadt, Germany) in 100% ethanol for 40 sec, 50% ethanol for 30 sec, 70% ethanol for 30 sec, 96% ethanol for 30 sec, 100% ethanol for 30 sec, and finally, twice in xylene for 5 min each. Slides were let to dry in the hood for 10 min and coverslipped.

To counterstain with 4′,6-diamidino-2-phenylindole dihydrochloride (DAPI), immunostained sections were not osmium intensified. After DAB-visualization of NOTCH1, sections were washed twice in 0.02 M KPBS for 5 min, and then, incubated for 10 min in the solution containing DAPI (1:1000, # 32670, Sigma-Aldrich, St. Louis, MO, USA) and 0.1% Triton X-100 in 0.02 M KPBS. Slides were washed once in 0.1 M PB and let to dry at RT. Finally, sections were coverslipped with ProLong Gold (#P36930, Life Technologies, Eugene, OR, USA).

To double-label with GFAP, acetone-fixed sections were first labeled with GFAP immunohistochemistry using a protocol described for NOTCH1 immunostaining above. As primary antibody, we used mouse monoclonal to GFAP (1:2000, #814369, Boehringer, Mannheim, Germany). As secondary antibody, we used Alexa488 goat anti-mouse antibody (1:500, #ab150117, Abcam). Slides were coverslipped with Vectashield (H-1000, Vector, Burlingame, CA, USA). Fluorescence-images were captured to visualize GFAP-immunohistochemical staining. Thereafter, the same sections were processed with NOTCH1 immunohistochemistry-protocol presented above without osmium intensification. Finally, sections were counterstained with hematoxylin and eosin (H&E) (#GHS216 and #HT110116, Sigma-Aldrich, St. Louis, MO, USA) to identify cytoarchitechtonics.

#### AQP4 and ANGPT1

Immunolabeling was performed using the same protocol as was described above for NOTCH1 (single labeling). Primary antibody used were mouse monoclonal antibody synthetic AQP4 (1:100, #ab9512, Abcam) and rabbit-polyclonal antibody raised against human ANGPT1 (1:200, ab102015, Abcam). Specificity of antibodies has been demonstrated previously [29–30].

### Assessment of NOTCH1 interactome protein expression

Expression of NOTCH1 protein was semi-quantitatively assessed from one section per rat (3 controls, 4 TBI) at a standardized coronal level, corresponding to the tissue sampling for molecular analysis (-3.4 mm from the bregma [31]). The number of NOTCH1-immunopositive cells in the whole dentate gyrus was counted in H&E counterstained sections, using a 40x objective field (see Fig 5E1 and 5E2). Thereafter, area of the region of interest (ROI) was measured with Image J software (version 1.50i, http://rsb.info.nih.gov/ij/) for each section. Finally, the density of immune-positive cells per mm^2^ was calculated.

To quantify the intensity of ANGPT1 immunoreactivity, digital RGB color images were captured from successive ANGPT1 -stained sections (-3.4 mm from the bregma [31]) using Leica DMRB microscope equipped with a Nikon DXM1200F camera operated by Nikon ACT-1 2.7 software. From each animal, 2–4 blood vessels were analyzed. In order to adjust the minimum and maximum gray scale values to be comparable to staining intensity in the original (RGB color) photomicrograph, RGB color images were converted to gray scale and thresholded (Image J software.) Blood vessels (with the cells) were outlined (ROI). Staining intensity inside the blood vessel was considered as background. The density of ANGPT1 immunolabeling was calculated by using the formula: (mean intensity of background ROI—mean intensity of ANGPT1)/mean intensity of background ROI. The mean intensity ANGPT1 staining in all vessels analyzed in a given animal was used for statistical analysis.

### Assessment of neuronal loss

To demonstrate that the downregulation of miRNA was not only due to the loss of neurons, we performed two different analyses. First, to assess the overall severity of neuronal damage in the dentate gyrus, we performed a visual analysis of thionin-stained preparations from rats (7 sham-operated and 10 TBI) perfused for histology at 3 months post-TBI (data not shown). Second, as inhibitory neurons are particularly vulnerable to lateral FPI-induced damage [32], we used the array data to assess the expression level of the neuronal marker, *Hrnbp3*, and particularly the markers of inhibitory neurons, parvalbumin (gene name: *Pvalb*), somatostatin (*Sst*), cholecystokinin (*Cck*), calretinin (*Calb2*), and neuropeptide y (*Npy*).

### Data analysis

The Exiqon Company performed the preliminary analysis of miRNA microarrays to identify upregulated and downregulated miRNAs. All calculations were performed with the software R/Bioconductor [33–34] primarily with the limma package. A P-value of less than 0.05 was considered statistically significant.

Analysis of the Affymetrix Rat Gene 1.1 ST arrays was performed using R/Bioconductor [35]. The microarrays were normalized with the Robust Multi-array Average algorithm (oligo package version 1.22.0) [36]. The intensity of the genomic probes below the median intensity of antigenomic probes (considered the background value) was corrected to this median value. Probes with an intensity equal to or lower than the background in more than five samples were removed from the analysis. Only probes that corresponded to a single gene were selected for further analysis. A one-way ANOVA was used to establish genes that were differentially expressed between groups. A P-value less than 0.05 was considered statistically significant.

To assess whether the different animal groups separated into their own clusters, we performed unsupervised hierarchical clustering in an R environment (version 3.0.1) [34] using the gplots package. Animals were ordered in a clustering heat map with the complete-linkage method together with the Euclidean distance measurement. Information about the cell type(s) expressing the miRNA of interest was revealed from the studies by McCall et al (36) and Jovicic et al [37]. Genomic location (RN5) of downregulated miRNAs (p<0.05) and upregulated or downregulated mRNAs (p<0.05) were investigated with the RCircos package in an R environment.

Targets of downregulated miRNAs were assessed using Ingenuity Pathway Analysis (IPA^®^) software (Ingenuity Systems, Redwood City, CA, USA) and Target Filter function. Only those mRNAs with a gene-expression change in the direction opposite that of its regulator miRNA were included for further analysis. For regulated mRNAs, predicted inhibition or activation of cellular processes were considered significant if p<0.05 and the prediction z-score >2.0 or <(-2) in the IPA^®^ Core analysis. Word Cloud Generator (https://www.jasondavies.com/wordcloud/) was used to visualize most common changes in categories of the cellular functions without z-score prediction. In the visualization, the spiral “Archimedean” and the scale “square root of n” were used.

Unsupervised hierarchical clustering (as mentioned above) was performed for miRNA targets to investigate possible gene clusters for specific molecular and biologic functions or cell types. Each of the formed clusters was analyzed independently with IPA^®^ Core Analysis without any cut-off values. For each miRNA target, a cell type known to be expressed was assessed from the database created by Zhang et al [38].

The STRING database [39] was used to study protein-protein interactions between miRNA targets. Cytoscape software [40] was used to convert STRING results to a simpler visual format. To further investigate the role of the NOTCH1 interactome in the dentate gyrus, NOTCH1 targets were analyzed using IPA^®^ Core Analysis. Predicted inhibition or activation of cellular processes was considered significant if the prediction z-score was >2.0 and p<0.05.

After qPCR, ddPCR and density measurement, a Mann-Whitney *U*-test (IBM SPSS Statistics 21) was used to assess differences between TBI animals and controls. A P-value less than 0.05 was considered statistically significant. For correlation analysis, Spearman’s rank was used (IBM SPSS Statistics 21).

For Gene Set Enrichment Analysis (GSEA), a pre-ranked gene list was created using the transcriptome profiling data containing 11,260 genes. Genes were stratified to negative and positive groups and ranked based on fold-change. Thereafter, the genes were arranged according to their p-value. Consequently, the higher the p-value, the closer to zero the gene was ranked. Downregulated genes are identified by the minus sign before the given p-value in the final list.

To combine all miRNA expression levels into a single parameter, factor analysis was performed to separate the principal components (IBM SPSS Statistics 21).

To check if upregulation of NOTCH1 interactome was replicated in another dataset, we analyzed sequencing results for the DG/CA3 and the perilesional cortex available in our laboratory [41].

## Results

### Mortality after lateral FPI

#### Animal cohort used for gene expression and miRNA analysis

In the sham-operated control group, acute mortality (<48 h post-surgery) was 8% (1/11). In the TBI group, acute mortality was 22% (4/18) (Fig 1) and late mortality (>48 h post-TBI) was 7% (1/14).

#### Animal cohort for immunohistochemistry

There was no mortality in the sham-operated or TBI animals (n = 10) (Fig 1).

### miRNA expression profiling revealed 12 downregulated miRNAs at 3 months post-TBI

**Fig 2:**
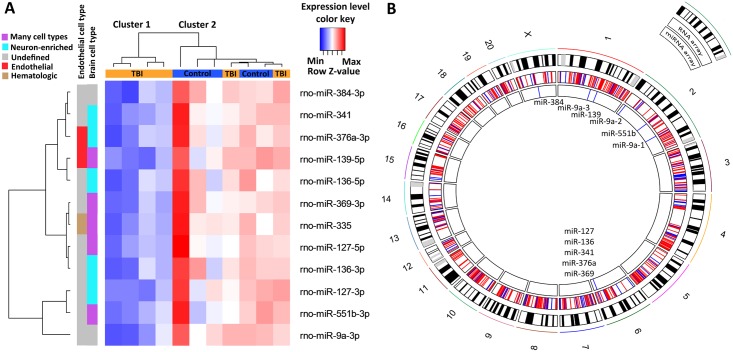
Expression change and genomic location of differentially regulated microRNAs (miRNAs) in the dentate gyrus at 3 months after Traumatic Brain Injury (TBI). **(A)** A heat map showing 12 downregulated miRNAs after TBI. Micro RNA names and the heat map color key are shown on the right side of the panel. Each column represents one animal and each row one miRNA. Data was clustered using Euclidean distance measurement. The dendrogram on the top shows the clustering of animals into two groups. Cluster 1 (left) contains 4 TBI rats and cluster 2 (right) contains all 5 controls and 2 TBI animals. Rats in cluster 1 have a clearly downregulated miRNA expression profile (lower expression indicated in blue) as compared to cluster 2. The dendrogram on the left side of the panel clusters the miRNAs into the groups. Color bars between the dendrogram and heat map indicate a cell type in which the particular miRNA is expressed according to previous experiments [36–37]. Purple color refers to miRNAs which under normal conditions are neuron-enriched even though they can be present also in other brain cell types (neurons, astrocytes, microglia or oligodendrocytes). (**B)** A circos plot showing the genomic location (rat genome version RN5) of downregulated miRNAs and mRNAs from the same samples. Of the 12 differentially expressed miRNAs, 7 (58%) belonged to two neuron-enriched clusters at chromosome 6 (Chr6q32). Abbreviations: Max, maximum row z-value; Min, minimum row z-value; TBI, traumatic brain injury.

Our profiling analysis at 3 months post-TBI revealed 12 downregulated mature miRNAs in the ipsilateral dentate gyrus (p<0.05; Fig 2A). Fold-changes in the miRNA levels were, from the smallest to the largest, as follows: 0.69 for rno-miR-369-3p, rno-miR-384-3p, rno-miR-136-5p, and rno-miR-127-3p; 0.71 for rno-miR-136-3p; 0.72 for rno-miR-335; 0.74 for rno-miR-376a-3p and rno-miR-551b-3p; 0.75 for rno-miR-341; 0.76 for rno-miR-139-5p; 0.77 for rno-miR-127-5p; and 0.82 for rno-miR-9a-3p. Assessment of the cellular expression of these miRNAs revealed that all but two are present in neurons, endothelial cells, or hematologic particles [34–35].

Unsupervised hierarchical clustering of the miRNA expression profile grouped 4 of the 6 TBI animals into their own cluster, whereas the remaining 2 TBI rats were placed into the same cluster as controls (Fig 2A). When downregulated miRNAs were plotted against the rat chromosomes (Fig 2B), five (miR-127, miR-136, miR-341, miR-376a, miR-369) belonged to two neuron-enriched non-coding RNA gene clusters in chromosome (Chr) 6, consistent with our cell type analysis (blue band in Fig 2A). MiR-9a has three genomic locations, one in Chr1 and two in Chr2. Further, a gene encoding miR-139 is located in Chr1, miR-551b in Chr2, and miR-384 in ChrX. Mir-335 does not have a known location in rat genome version 5.

### IPA^®^ predicted 75 upregulated targets for 6 of the 12 downregulated miRNAs

Messenger RNA expression profiling with microarrays revealed 654 upregulated and 212 downregulated genes at 3 months post-TBI (p<0.05) (Fig 2B). Differentially expressed genes were located in all chromosomes, although some chromosomal areas seemed to be avoided (*e*.*g*., in Chr4 or Chr6; Fig 2B).

**Fig 3:**
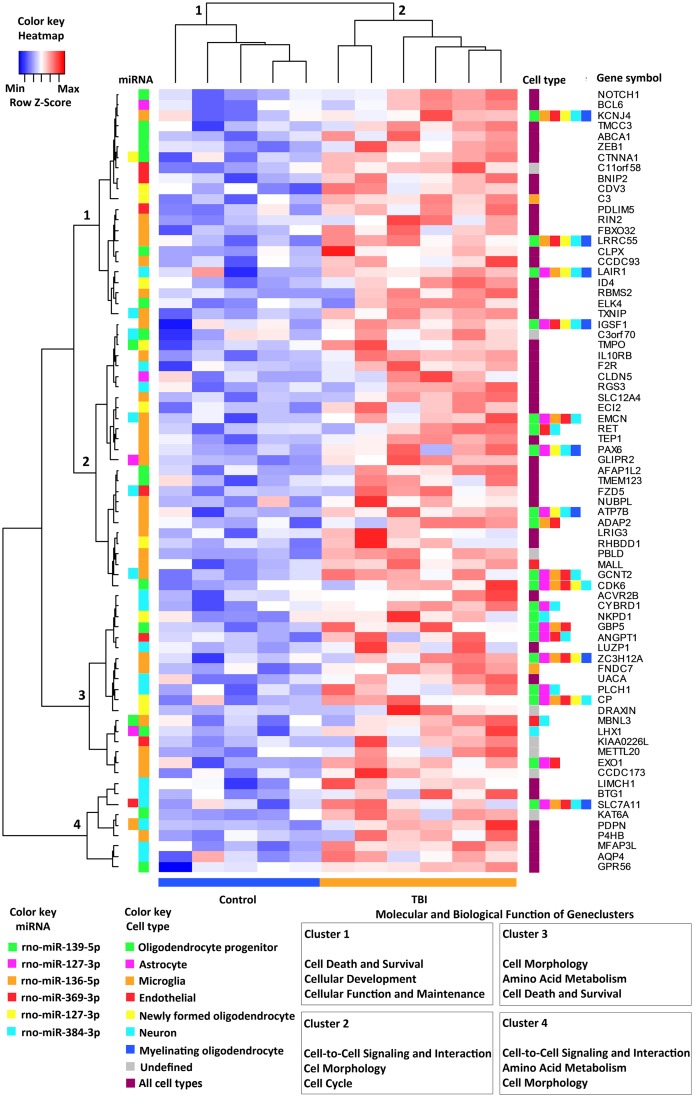
A clustering heat map specifying the 75 upregulated genes that were targeted by downregulated miRNAs. In the heat map, each column represents one animal and each row one gene product. A dendrogram above the heat map separates TBI animals (cluster 1) from controls (cluster 2). Cell types [36] expressing the gene products are shown on the right side of the heat map. No clustering was observed according to the cell type. The regulating miRNAs, analyzed by IPA^®^ Target Filter, are indicated on the left side between the heat map and dendrogram. Some mRNAs were regulated by two different miRNAs at the same time. The dendrogram on the left divides miRNA targets into four separate clusters. When clusters were analyzed with IPA^®^ Core Analysis, a different molecular and biologic function was found for each of clusters 1–4 (lower right corner of the panel). Abbreviations: Max, maximum row z-value; Min, minimum row z-value; miRNA, microRNA; TBI, traumatic brain injury.

The IPA^®^ Target filter predicted a total of 75 targets (experimentally validated, high or moderate prediction) for 6 different miRNAs (Fig 3). For the remaining 6 miRNAs, the IPA^®^ database was unable to identify a target. From the identified mRNA targets, 16% (12 of 75) were regulated by 2 different miRNAs (Fig 3). Mir-384-3p was present in 7 miRNA pairs, miR-136-5p in 6 pairs, miR-139-5p in 5 pairs, and the others (miR-127-3p, miR-127-5p, and miR-369-3p) in 2 pairs. Interestingly, miR-384-3p was targeted together with miR-136-5p 4 times and with miR-369-3p 2 times. The upregulation of target mRNA by miRNA pairs occurred most often in endothelial cells (50%, 6 of 12 mRNAs).

When analyzed in IPA^®^ with z-score prediction, the cellular function categories among the regulated mRNAs were organismal survival, developmental disorder, cell death and survival, hematologic system development and function, tissue morphology, gene expression, cell-to-cell signaling and interaction, organ morphology, reproductive system development and function, and molecular transport (S3 Table).

Unsupervised hierarchical clustering did not separate miRNA targets by the regulator (*i*.*e*., a particular miRNA) or by the cell type, in which the gene was reported to be most commonly expressed (Fig 3). When gene clusters were analyzed individually (clusters of the dendrogram on the left side of Fig 3) using IPA^®^, we found four clusters with slightly different molecular and cellular functions. The functions of cluster 1 were cell death and survival, cellular development, and cellular function and maintenance. The cluster 2 functions included cell-to-cell signaling and interaction, cell morphology, and cell cycle; cluster 3 functions included cell morphology, amino acid metabolism, and cell death and survival; and cluster 4 functions included cell-to-cell signaling and interaction, amino acid metabolism, and cell morphology.

### Bioinformatics analysis highlighted upregulation of transcription factor *Notch1*, a target for miR-139-5p

**Fig 4:**
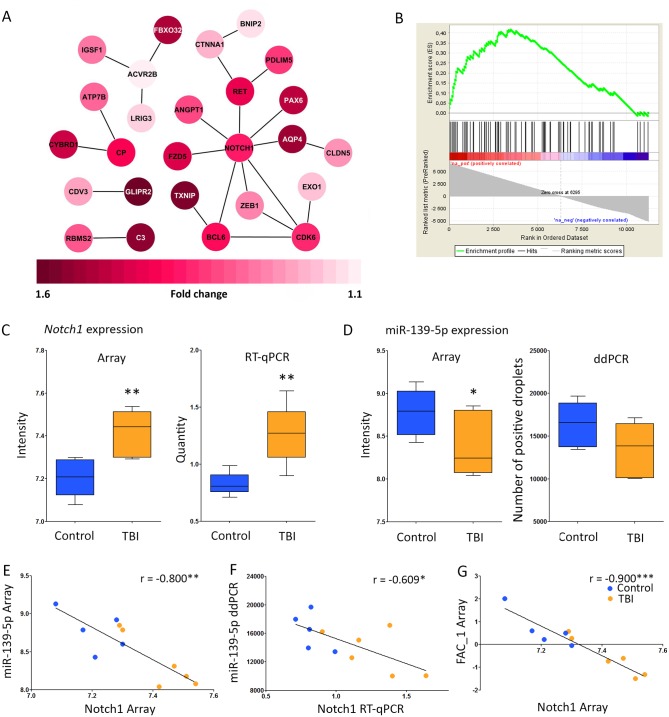
Downregulation of mir-139-5p was associated with an upregulation of the transcription factor *Notch1*. **(A)** STRING analysis revealed that NOTCH1 had the most abundant protein-protein interactions with other miRNA-regulated proteins (8/23 interactions). For clarity, proteins without any interactions with other miRNA targets were omitted from the figure. Red color-coding indicates upregulated genes (1.6–1.1-fold as compared to controls, p<0.05). Darker the red color, more upregulated the gene expression. **(B).** Gene Set Enrichment Analysis (GSEA) showed positive enrichment of 48% (39/82) known (IPA^®^ database) NOTCH1 target genes in the mRNA microarray (Rank numbers 1-3448/11260, FDR<0.01, enrichment score 0.42), suggesting activation of NOTCH1 protein. **(C)** Upregulation of *Notch1* gene expression on the microarray (fold-change 1.16, p<0.01) was replicated by quantitative reverse transcription PCR (RT-qPCR; fold-change 1.53, p<0.01). **(D)** Downregulated miR-139-5p expression on the microarray (fold-change 0.76, p<0.05). Analysis of miR-139-5p expression with droplet digital PCR (ddPCR) revealed a trend towards downregulation (fold-change 0.83). **(E)** *Notch1* and miR-139-5p expression levels were tightly correlated on array analysis (r = -0.80, p<0.01), and importantly, also in **(F)** PCR replication analysis (r = -0.61, p<0.05). **(G)** Factor analysis was used to combine the microarray intensity for all 12 downregulated miRNAs to reduce the data, and analysis of the first principal component to *Notch1* expression led to an even better correlation (r = 0.9, p<0.01) than for miR-139-5p alone. This indicates that one or more other miRNAs regulates the expression of *Notch1*, either directly or indirectly via their target genes. Statistical significance: *, p<0.05; **, p<0.01; ***, p<0.001. Abbreviations: ddPCR, droplet digital PCR; FAC_1, 1^st^ principal component from the factor analysis; TBI, traumatic brain injury; r, correlation coefficient; RT-qPCR, quantitative reverse-transcription PCR.

To pinpoint the major miRNA-regulated networks at the chronic post-TBI time point, we complemented the IPA^®^ pathway analysis with a GSEA and STRING protein-protein interaction analysis of the miRNA targets. These analyses highlighted the NOTCH1 pathway both on the list of miRNA-targeted genes (Fig 4A) as well as on the list of all upregulated or downregulated genes on the mRNA microarray (Fig 4B). The STRING interaction analysis showed that NOTCH1 had 8 known direct and 6 known indirect contacts with other miRNA target genes (Fig 4A). The GSEA analysis indicated that from the 82 known (IPA^®^ database) NOTCH1 target genes present in our microarray dataset, 48% (39/82) were enriched in the pre-ranked microarray data (Fig 4B, Rank numbers 1-3448/11260, FDR<0.01, enrichment score 0.42). These data strongly suggested long-lasting activation of NOTCH1 after TBI.

To further assess the role of upregulated NOTCH1, we performed an IPA^®^ Core Analysis for enriched NOTCH1 targets (a list from the GSEA analysis). Those gene products were most often involved in enhanced organism injury and abnormalities, as well as in different cellular functions, such as cellular development, growth and proliferation, and movement (S4 Table). Interestingly, up to 59% (23/39) of the enriched NOTCH1 targets were involved in transcription (S4 Table), *e*.*g*., increasing the transactivation of RNA, transcription and expression of RNA, and binding to protein binding site; or activation of the DNA endogenous promoter.

### Validation of bioinformatics analysis confirmed the increased level of NOTCH1 mRNA and activated protein in the dentate gyrus at 3 months post-TBI

We then confirmed the expression of *Notch1* and its regulator, miR-139-5p [42–44]. Quantitative RT-PCR confirmed the upregulation of *Notch1* (p<0.05, Fig 4C). Droplet dPCR analysis showed a trend toward downregulation of rno-miR-139-5p (Fig 4D), consistent with the array analysis (Fig 2A). Importantly, *Notch1* gene expression was correlated with rno-miR-139-5p gene expression in both the gene array analysis (r = -0.800, p<0.01) and the replicate PCR analyses (r = -0.609, p<0.05; Fig 4E and 4F). When we took the 1^st^ component from the principal component analysis of all 12 downregulated miRNAs on the array, and analyzed their correlation to *Notch1* gene expression, we obtained a remarkably higher correlation coefficient than with rno-miR-139-5p alone (r = -0.900, p<0.001, Fig 4f). These data indicate that other miRNAs downregulated at 3 months post-TBI may also regulate *Notch1* gene expression either directly or indirectly via their target genes.

**Fig 5:**
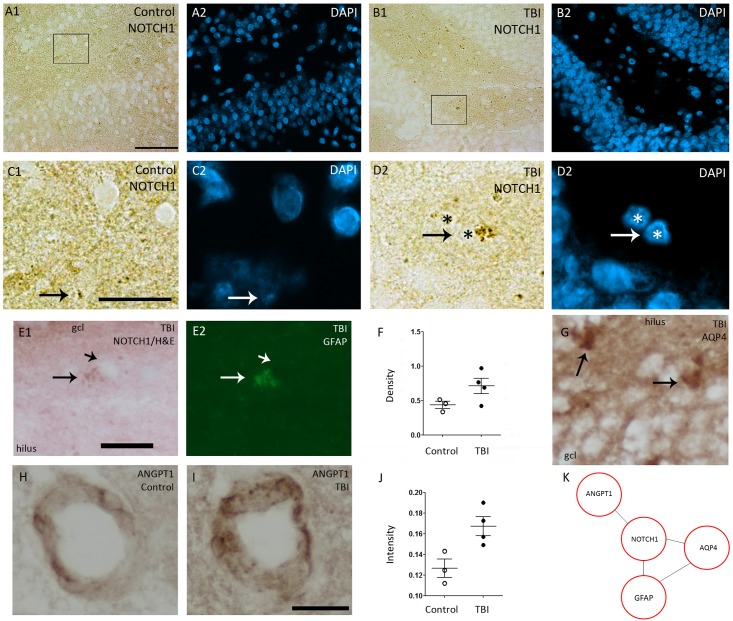
Representative photomicrographs showing the expression of NOTCH1 interactome in the dentate gyrus at 3 months post-TBI. A sham-operated control showed **(A1)** light immunostaining of NOTCH1 **(A1)**. After TBI **(B1)** strong immunostaining of NOTCH1 was observed in all layers of the dentate gyrus. Panels **A2** and **B2** show DAPI staining in representative sections. A higher magnification image of **(C1)** NOTCH1 and **(C2)** DAPI from a boxed area in panel A1. Arrows point to an immunopositive cell. Higher-magnification images of **(D1)** NOTCH1 and **(D2)** DAPI from a squared area in panel B1. Note an increased intracellular (particularly cytoplasmic) NOTCH1 immunoreactivity (arrows) after TBI as compared to the cell in panel C1 from a control animal. Asterisks indicate the same nuclei in D1 and D2. **(E1)** NOTCH1 immunoreactive cell in a Hematoxylin & Eosin counterstained section in a rat with TBI (arrow). Short arrow points to nucleus. **(E2)** The same section counterstained with GFAP showing that the NOTCH1 positive cell is an astrocyte. **(F)** Dot blots showing a trend towards increased density of NOTCH1 immunopositive cells in the dentate gyrus of rats with TBI (p = 0.149). **(G)** AQP4-immunopositive cells (arrows) in the dentate gyrus after TBI. ANGPT1-immunopositive blood vessels in **(H)** control and **(I)** TBI rat. **(J)** Quantification indicated a trend towards increased intensity of ANGPT1-immunostaining after TBI (p = 0.057). **(K)** A summary of NOTCH1 interactome showing an upregulation at protein level after TBI. Abbreviations: gcl, granule cell layer; mol, molecular layer; TBI, traumatic brain injury. Scale bar equals 30 μm (panels A-B), 10 μm (panels C-G) and 20 μm (panels H-I).

To validate the bioinformatics prediction of NOTCH1 activation, we performed immunostaining for the Val1744-cleaved form of NOTCH1, *i*.*e*., activated NOTCH1, which is also called NICD. In the ipsilateral dentate gyrus of sham-operated controls, we observed only a very light NICD immunostaining, and the number of immunopositive cells per section varied between 47 to 63 (Fig 5A, 5C and 5F). In all four rats with TBI, number of positive cells was increased ipsilaterally, varying between 54 to 117 (Fig 5B, 5D and 5F). Double-labeling revealed high post-TBI NICD immunoreactivity in GFAP-positive astrocytes (Fig 5E). Our analysis of post-TBI upregulation of several components of Notch1 “interactome” revealed cellular upregulation of AQP4 in the infragranular region of the dentate gyrus, thus corresponding to the localization of Notch1+ cells (Fig 5E and 5G). We also found an increased vascular ANGPT1 staining in the dentate gyrus (Fig 5h and 5I) after TBI (Fig 5J).

Finally, we assessed whether the expression of *Notch1* gene would differ between post-TBI animals and controls in the perilesional cortex [39]. Our data show that *Notch1* gene expression in the perilesional cortex was 1.6-fold after TBI (FDR<0.001). In the perilesional cortex, we also found upregulated (commonly referred as NOTCH1 targets) *Hey1*, *Hey2* and *Hes5* (1.66, FDR<0.001; 1.33, FDR<0.05; 1.30, p<0.05; respectively).

### Association of neuronal loss with changed transcription

**Fig 6:**
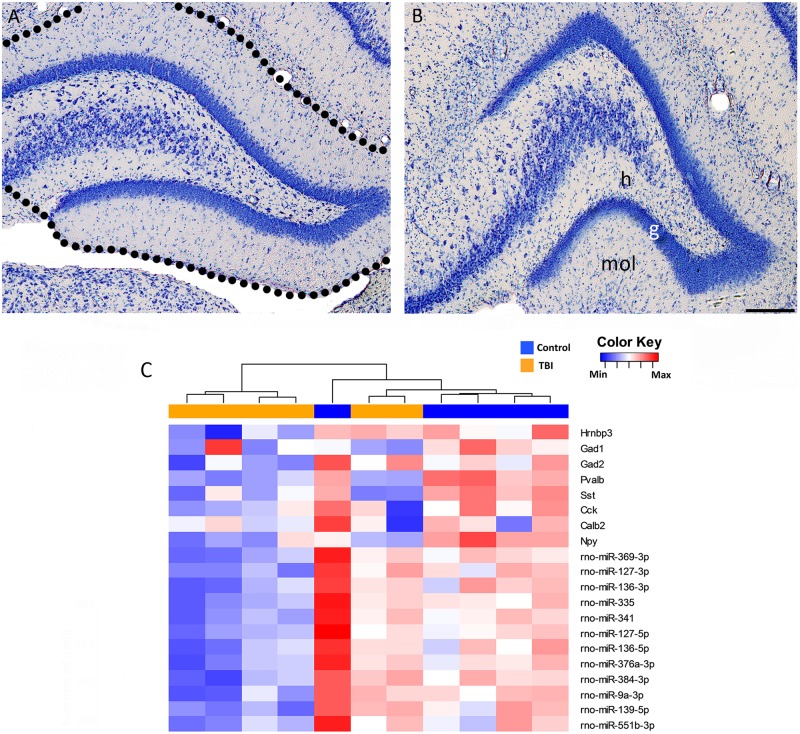
Neuronal loss after Traumatic Brain Injury (TBI). Representative photomicrographs of a thionin-stained section from the dentate gyrus of **(A)** a sham-operated control rat and **(B)** a rat with lateral fluid-percussion induced (FPI) TBI 3 months earlier. Note that the principal cells located in the granule cell layer and proximal CA3c are well preserved. The loss of hilar neurons, however, was remarkable. Dashed outline shows the dissected tissue used for mRNA and microRNA array analysis. **(C)** A heat map presenting the gene expression of neuronal marker *Hrnbp3* and 7 interneuronal markers (*Gad1*, *Gad2*, *Pvalb*, *Sst*, *Cck*, *Calb2*, and *Npy*) as well as the expression of the 12 downregulated miRNAs. Two rats with TBI clustered together with the controls. Expression of the *Hrnbp3* gene in these animals was not changed, whereas the gene expression of interneuronal markers was remarkably decreased. Abbreviations: g, granule cell layer; h, hilus; Max, maximum; Min, minimum; mol, molecular layer; TBI, traumatic brain injury.

Next, we considered the possibility that downregulation of miRNAs was related to TBI-induced neuronal loss. As shown in representative thionin-stained sections from a control rat (Fig 6A) and a rat 3 months after TBI (Fig 6B), the granule cells remained relatively well preserved. Consistent with our previous stereologic analysis of inhibitory neurons in the dentate gyrus [30], expression of interneuronal markers was remarkably downregulated in the gene expression analysis (Fig 6D).

## Discussion

The present study investigated brain miRNA expression in chronic post-TBI to test the hypothesis that transcriptional alterations in the dentate gyrus are under chronic miRNA control. We had three major findings. First, 12 miRNAs were downregulated, and 6 of the 12 belonged to a Chr6 non-coding RNA cluster. Second, the major biologic functions related to the miRNA-targeted mRNAs were cell death and survival, hematologic system development and function, tissue morphology, gene expression, cell signaling, and molecular transport. Third, and most importantly, our analysis revealed that the NOTCH1 interactome is a novel chronically up-regulated miRNA controlled pathway.

### Many chronically downregulated miRNAs belong to the Chr6 non-coding RNA gene cluster and modulate biologic processes linked to post-TBI morbidities

Our miRNA array study showed the downregulation of 12 miRNAs at 3 months post-TBI. Interestingly, 4 of the 6 TBI animals had a clearly downregulated miRNA profile, whereas the two remaining animals were comparable to controls, supporting the previously reported variability between animals in the model used [25–26, 32]. Interestingly, 7 of the 12 downregulated miRNAs belonged to a Chr6 non-coding RNA gene cluster. This cluster is regulated by epigenetic alterations, including histone modifications and DNA methylation [45]. Our recent DNA methylation analysis in the dentate gyrus at 3 months post-TBI, however, revealed no changes in the methylation status of the regulatory or gene areas of these 12 down-regulated miRNAs [46]. Whether post-TBI miRNA expression is controlled by histone modifications remains to be explored. In particular, recent data show that histone deacetylase inhibitors alleviate hippocampal neurodegeneration and counteract the development of hippocampus-dependent memory deficits after TBI [47–48]. Moreover, chromatin-modifying drugs restore the expression of specific miRNAs such as miR-127, which was downregulated in the present study [45].

**Fig 7:**
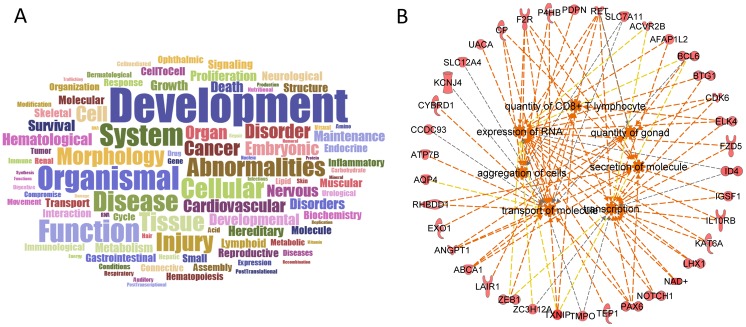
Data summary. Bioinformatics analysis of miRNA targets highlighted a variety of pathologies of the dentate gyrus after TBI. **A.** A word cloud representing the most common words among the miRNA-targeted biologic functions. The top words were development > organismal > function > system > cellular > disease > abnormalities > tissue > injury. **B.** IPA^®^ Core analysis predicted that many of the biologic functions previously connected to dentate gyrus pathologies were upregulated, including secretion of molecules, transcription, expression of RNA, and quantity of CD8+ T lymphocytes. Orange line, leads to activation; yellow line, findings inconsistent with the state of the downstream molecule; gray line, effect not predicted.

An IPA^®^ core analysis was performed to investigate the cellular functions of upregulated miRNA mRNA targets and is summarized in Fig 7. The most targeted functions included development > organismal > function > system > cellular > disease > abnormalities > tissue > injury. These miRNA-regulated functions align well with chronic functional impairments associated with TBI-induced pathology in the dentate gyrus. For example, IPA^®^ analysis of miRNA targets converged to a functional gene ensemble called “secretion of molecule” which regulates neurogenesis in the dentate gyrus [49]. Interestingly, miR-139-5p and miR-335, which were identified in our analysis, also regulate neurogenesis [50–51]. “Transcription” or “expression of RNA” regulate spatial memory and/or long-term potentiation [49], which are regulated by miR-127-5p, miR-136-5p, and miR-376a-3p [52–53]. Finally, “quantity of CD8+ T lymphocyte”, “secretion of molecule”, and “transcription” are associated with temporal lobe epilepsy [54–57], in which the expression of miR-9a and miR-139-5p are altered [58–59].

### Upregulation of NOTCH1 by downregulation of selective miRNAs after TBI

To better understand the mechanisms by which miRNA downregulation chronically modulates the molecular pathways contributing to functional outcomes after TBI, we performed an extensive bioinformatics analysis of the mRNA and miRNA array datasets. Messenger RNA expression profiling with a microarray revealed 654 upregulated and 212 downregulated genes at 3 months post-TBI compared with controls, and the changes were distributed throughout the whole rat genome. The STRING analysis indicated that of all 75 upregulated miRNA targets in our study, NOTCH1 has the best known and predicted protein-protein interactions. Moreover, IPA^®^ miRNA Target Filter analysis predicted that *Notch1* mRNA is targeted by miR-139-5p. This was confirmed in our microarray study, showing a downregulation of miR-139-5p in the dentate gyrus at 3 months post-TBI. Moreover, ddPCR analysis showed a trend toward a decreased level of miR-139-5p in the same sample. In parallel, levels of *Notch1* mRNA were elevated and the protein level of activated NOTCH1 was increased. Our data are consistent with those of previous experimental studies showing regulation of *Notch1* mRNA by miR-139-5p [42–44, 60]. Moreover, both miR-139-5p [43] and the 3’ untranslated region of the *Notch1* [41] appear well-conserved among mammals, further emphasizing the true regulative potential of miR-139-5p on *Notch1* mRNA.

Notch signaling is found from stem cells to mature neurons, and even in degenerating neurons [61]. In the adult brain, Notch signaling has been argued to work as a master regulator of synaptic and dendritic plasticity (reviewed by [61]). Compared with other members of the Notch protein family, NOTCH1 signaling appears to be important for different aspects of the repair processes of the adult brain [62]. In particular, NOTCH1 signaling is linked to synaptic plasticity [63], angiogenesis and blood vessel morphogenesis [64], postnatal lymphatic development [65], neurogenesis [66], differentiation and development of reactive astrocytes [67–68], dendritic arborization and spine formation [69], and endocytosis [70]. Not surprisingly, dysregulation of NOTCH1 signaling has been mechanistically linked to the dentate gyrus- and hippocampus-related pathologies present after TBI, including epilepsy via gliosis [71–72], stress and depression via neurogenesis [66, 73–74], and learning and memory deficits via synaptic plasticity [74].

The little previous data available on hippocampal NOTCH1 changes after TBI comes from studies performed at acute time-points. Zhang et al [60] reported a downregulation of *Notch1* mRNA and protein at 2 h after lateral FPI and suggested that this could trigger the activation of Ngn2, and consequently, neuronal differentiation. Wang et al [75] profiled miRNA expression at 3 d post-controlled cortical impact-induced TBI in the dentate gyrus and reported downregulation of miR-34a and upregulation of *Notch1* mRNA and protein. They also reported that low temperature treatment decreased *Notch1* mRNA, which was related to normalized miR-34a levels. The protein level, however, remained elevated. NOTCH1 has been reported to change also in other brain injury models, including pilocarpine and intrahippocampal kainic acid models of status epilepticus (SE) [66, 71–72]. In human temporal lobe epilepsy, patients with hippocampal sclerosis have higher NOTCH1 expression than patients without hippocampal sclerosis [71–72]. In these conditions, NOTCH1 signaling abnormalities are linked to disrupted dendritic arborization, increased number of dendritic spines, astrogliosis, and altered neurogenesis. Functional links to the evolution of epileptogenesis and seizure occurrence due to the abnormal formation of neuronal circuits have also been demonstrated [16,32].

Taken together, several lines of evidence support the idea that upregulation of *Notch1* compromises the recovery process in the damaged hippocampus and dentate gyrus, which is linked to an unfavorable outcome, including epileptogenesis. The lowering of NOTCH1 levels via therapeutic interventions has shown some promise. Zileuton, a 5-lipooxygenase inhibitor administered after pilocarpine-induced SE [71] or N-[N-(3, 5-difluorophenacetyl)-L-alanyl]-S-phenylglycine t-butyl ester after kainic acid-induced SE [72] reduced NOTCH1 levels and alleviated the epileptogenesis. Finally, low-temperature treatment after TBI reduced NOTCH1 upregulation by dampening the downregulation of miRNA-34a [76]. Interventions that would specifically alter NOTCH1-regulating miRNAs could potentially provide appealing means to modify post-injury outcome. Promising data were reported by Qu et al. [77], who demonstrated that an agomir targeting miR-139-5p, a miRNA that was regulated in the present study at 3 months post-TBI, attenuated brain damage when administered at 12 h after hypoxic-ischemic injury. Whether activation of the NOTCH1 pathway contributed to the favorable outcome and which were the brain cell type(s) upregulating NOTCH1 post-TBI remain to be studied.

### Cellular location of miRNA expression in the dentate gyrus after TBI

The dentate gyrus of the adult rat contains ~1.1 million granule cells and 50 thousand hilar neurons [78]. Previous stereologic analysis revealed that lateral FPI results in relatively mild damage to the principal cells in the dentate gyrus, as on average only ~10% of granule cells, the prevailing cell type, are lost [78]. The loss of hilar neurons is remarkable (up to 50%), including a 50% to 60% loss of different subclasses of inhibitory neurons [7, 32]. The pattern of neurodegeneration in sections available for histologic analysis in the present analysis was comparable to that presented previously.

As our bioinformatics analysis revealed that 10 of the 12 downregulated miRNAs are expressed in neurons, we were concerned that the reduction in miRNA expression could be related to neuronal loss rather than to transcriptional downregulation. The heat map analysis of different cell type markers revealed that two of the TBI animals that clustered together with controls had no loss of *Hrnbp3* mRNA, a gene encoding a neuronal marker NeuN, and on average its expression did not differ between the injured and control animals. Thus, the normal levels of *Hrnbp3* correspond well with the histologic analyses that discussed the large number of granule cells that remain. Each injured rat showed a decrease in interneuronal markers, corresponding to a remarkable loss of hilar interneurons, including parvalbumin, neuropeptide Y, somatostatin, cholecystokinin, and calretinin in the lateral FPI model [32].

Despite the robust interneuron loss suggested by the expression analysis, the expression of target genes for the 12 downregulated miRNAs was upregulated in the TBI animals. This is of particular interest as 5 of the 12 downregulated miRNAs (miR-127-3p, miR-127-5p, miR-136-3p, miR-136-5p, and miR-384) are expressed in higher levels in inhibitory than in excitatory neurons [79]. In particular, 4 of 12 downregulated miRNAs (miR-335, miR-341, miR-139-5p, miR-551b-3p) are expressed in high levels in somatostatin-positive interneurons, of which only 46% remain in the injured dentate gyrus (1 month time-point [32]). Only miR-335 expression is reported to be more abundant in excitatory than inhibitory neurons [79]. If the downregulation of miRNAs was related only to the loss of inhibitory neurons, we would expect the expression of miRNAs targeting mRNAs to be downregulated as well, which was not the case. Therefore, our interpretation is that the downregulation of miRNAs detected in this study was related to a change in miRNA gene expression or processing rather than solely to neuronal loss.

### Chronically downregulated miRNAs are also regulated acutely after TBI as well as after other types of experimental and human hippocampal damage

How long does the downregulation of the 12 miRNAs last? A literature search revealed that some of the 12 miRNAs that were downregulated at 3 months are regulated already at the acute post-TBI phase. Redell and colleagues [14] performed a miRNA microarray analysis the whole hippocampus at 3 and 24 h after controlled cortical impact and reported downregulation of miR-127 and miR-376a at 3 h post-TBI. They also reported an upregulation of miR-9, which was, however, downregulated in our study at 3 months post-lateral FPI. Liu et al [15] induced controlled cortical impact injury and analyzed miRNA expression in the whole hippocampus at 1 h, 2 h, 3 d, 5 d, or 7 d post-TBI using microarrays. None of the miRNA changes reported matched those found in the present study. Interestingly, we found no sex differences in the post-TBI miRNA expression. Taken together, these data suggest that some expression changes in miRNA can be present both at acute and chronic post-TBI phases, independent of the injury model.

We then explored whether the chronic hippocampal miRNA profiles after lateral FPI would compare to those in which hippocampal neuropathology is qualitatively similar, e.g., neurodegeneration, neurogenesis, gliosis, synaptic and axonal plasticity, and vascular changes, but the injury in the dentate gyrus was induced by different means. Hu et al [80] induced SE with Li-pilocarpine and profiled miRNA expression using microarrays at 2 months post-SE. Similar to our study, they found a downregulation of miR-136. Recently, Gorter et al. [81] performed microarray miRNA profiling in the dentate gyrus at 3–4 months after hippocampal stimulation-induced SE in adult male Sprague Dawley rats. As in our study, they found that miR-139-5p was downregulated, although the significance of the data was not specified. Several other studies analyzing hippocampal miRNAs in SE models between 3 weeks to 3 months post-injury did not reveal any similarities to the present data [82–84]. These studies suggest that regulation of some hippocampal miRNAs after injury can be model-independent, but there are also major model-dependent differences.

Regarding the translational value of our data, we next compared the miRNA expression in the present study to that in human injured hippocampus. Kan et al. [85] reported 165 dysregulated miRNAs in hippocampi resected from patients with drug-refractory temporal lobe epilepsy. Interestingly, miR-139-5p was downregulated in a subgroup of patients with hippocampal sclerosis compared with controls. miR-551b was downregulated and miR-9 upregulated in all epilepsy patients compared with controls. McKiernan et al [19] reported mainly downregulation (51% of all miRNAs expressed) of miRNAs in the patient group with temporal lobe epilepsy and hippocampal sclerosis. Similar to our study, miR-335 was downregulated.

To summarize, downregulation of miR-139-5p appears as a common chronic change in damaged dentate gyrus, independent of whether the damage was induced by lateral FPI or by electrical stimulation of the perforant pathway in rats, or sclerotic hippocampus in humans. Although controlled comparative studies remain to be done, the data suggest some long-lasting, model-independent changes in miRNA regulation. Also, the biologic and molecular pathways targeted by the altered miRNAs were strikingly similar at the acute and chronic phases after TBI, as well as in chronic samples analyzed in different experimental models and human tissue.

## Conclusion

The miRNA expression profile in the dentate gyrus remains chronically downregulated after TBI and is associated with a long-lasting upregulation of target genes. Bioinformatics analysis and wet-lab validation highlighted an elevation in the transcription factor NOTCH1 as a miRNA target, which has a potential to modify the evolution of secondary brain injury as well as tissue recovery by regulating the expression of genes necessary for angiogenesis, astrogenesis, and neurogenesis. As the transcriptional regulation by miRNAs was clear even at 3 months post-TBI, our data suggest that miRNA-targeted interventions could modulate dentate gyrus-related pathologies over a wide therapeutic time window. However, there is a further need to explore the specific NOTCH1 regulating miRNA pathways in different cell types, and their potential to favorably modulate pathologies at the given post-injury time point, for example, by silencing of specific miRNAs *in vitro* and/or *in vivo* to direct candidate therapies to the right cells at the right post-TBI time point.

## Supporting information

S1 Table miRNA expression changes after TBI. Spreadsheet containing results after statistical analysis.
(XLS)

S2 Table Gene expression changes after TBI. Spreadsheet containing results after statistical analysis.
(XLS)

S3 Table Cellular function categories among the regulated mRNAs. Spreadsheet of results exported from IPA.
(XLS)

S4 Table Diseases or functions annotation for NOTCH1 targets. Spreadsheet of results exported from IPA.
(XLS)
